# Supplementary material for: Genomic Characterization of Parengyodontium torokii sp. nov., a Biofilm-Forming Fungus Isolated from Mars 2020 Assembly Facility
Source: J Fungi (Basel). 2022 Jan 9;8(1):66. doi: 10.3390/jof8010066 (PMC8778116; doi:10.3390/jof8010066)
Supplement: Supplementary file 1 [file jof-08-00066-s001.zip › jof-1493606-supplementary.pdf]

**Supplemental Figure Legends.**

**Figure S1. Scanning electron micrography of *Parengyodontium torokii* CBS 368.72 strain. (A)** Zigzag rachides of the fertile conidiogenous cells. **(B)** Subcylindrical to ellipsoidal, hyaline single-celled conidia.

**Figure S2. Molecular phylogenetic analysis of the ITS region from *Parengyodontium* spp. and related taxa in the Cordycipitaceae.** Phylogram of the most likely tree (−lnL = 1622.37) from a ML analysis of 24 sequences based on the ITS region (568 bp) using IQ-TREE. Numbers refer to UFBootstrap support values ≥ 90% based on 5000 replicates. Nodes ≥95 are considered strongly supported. Bayesian posterior probabilities ≥ 95% are denoted as thickened branches. *Isaria coleopterora* (CBS 110.73) is used outgroup. ET designates ex-type isolates. Newly described species is in bold and highlighted. Bar indicates nucleotide substitutions per site.

**Figure S3. Molecular phylogenetics of concatenated loci, two ribosomal (ITS and LSU) and one protein-coding locus (β-tubulin) from *Parengyodontium* spp. and related taxa in the Cordycipitaceae.** Phylogram of the most likely tree (−lnL = 3824.45) from a ML analysis of 22 sequences based on the combined regions of ITS, LSU and TUB (1435 bp) using IQ-TREE. Numbers refer to UFBootstrap support values ≥ 90% based on 5,000 replicates. Nodes ≥95 are considered strongly supported. Bayesian posterior probabilities ≥ 95% are denoted as thickened branches. *Isaria coleopterora* (CBS 110.73) is used outgroup. ET designates ex-type isolates. Newly described species is in bold and highlighted. Bar indicates nucleotide substitutions per site. Phylogram of the most likely tree (−lnL = 1622.37) from a ML analysis of 24 sequences based on the ITS region (568 bp) using IQ-TREE. Numbers refer to UFBootstrap support values ≥ 90% based on 5000 replicates. Nodes ≥95 are considered strongly supported. Bayesian posterior probabilities ≥ 95% are denoted as thickened branches. *Isaria coleopterora* (CBS 110.73) is used outgroup. ET designates ex-type isolates. Newly described species is in bold and highlighted. Bar indicates nucleotide substitutions per site.

**Figure S4. Biofilm structures of *P. torokii* FJII-L10-SW-P1 strain. (A)** A thick layer of biofilm mat containing conidial spores and fungal mycelia. White arrow is pointing the sheath of fungal biofilm mat. **(B)** Arrow pointing to the white material where conidial spores are positioned. **(C)** Arrow pointing to a pair of conidia that were attached to the white fibrous material. **(D)** A thin sheet of fungal biofilm mat containing only conidia. Note only conidial spores are associated with these structures and not fungal mycelia.

**Figure S5. Mitogenome of *Parengyodontium torokii*.** The circular map of the mitochondrial genome of *P. torokii* was drawn with OGDRAW and core genes related to the electron transport chain, ribosomal protein, transfer RNAs, putative additional ORFs and introns are displayed.

**Figure S6. Genomic annotations of secreted proteins among Cordycipitaceae species.** Total number of predicted secreted proteins for each individual species were plotted using bar graphs**.**

**Figure S7. Comparative genomic analysis of predicted Secondary Metabolite Cluster among 9 Cordycipitaceae genomes.** The secondary metabolite clusters were predicted and annotated using the antiSMASH 6 pipeline. All predicted clusters were countered and plotted in a heatmap.

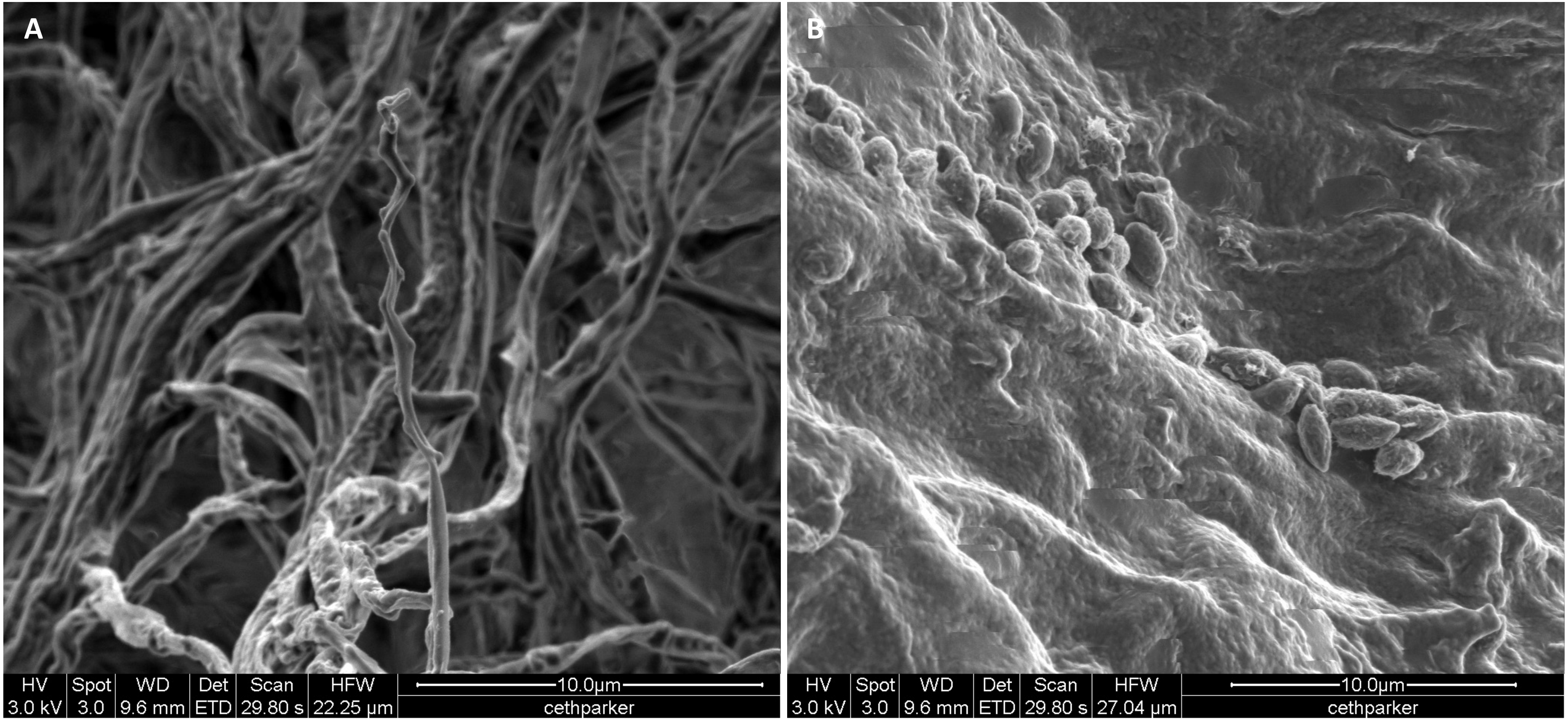


**Figure S1. Scanning electron micrography of *Parengyodontium torokii* CBS 368.72 strain. (A)** Zigzag rachides of the fertile conidiogenous cells. **(B)** Subcylindrical to ellipsoidal, hyaline single-celled conidia.

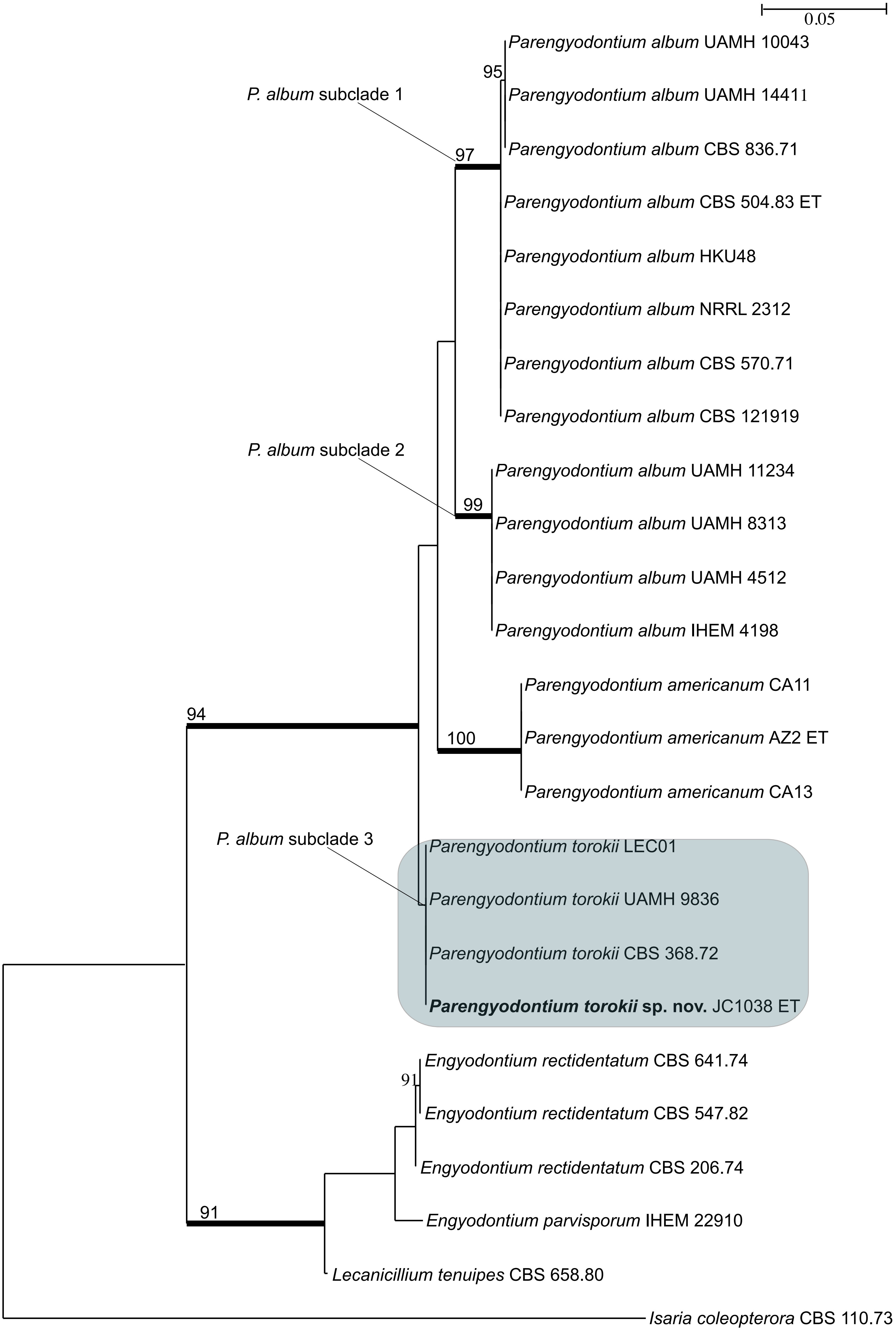
0.05
Parengyodontium album UAMH 10043
95
Parengyodontium album UAMH 14411
P. album subclade 1
97
Parengyodontium album CBS 836.71
Parengyodontium album CBS 504.83 ET
Parengyodontium album HKU48
Parengyodontium album NRRL 2312
Parengyodontium album CBS 570.71
Parengyodontium album CBS 121919
P. album subclade 2
Parengyodontium album UAMH 11234
99
Parengyodontium album UAMH 8313
Parengyodontium album UAMH 4512
Parengyodontium album IHEM 4198
Parengyodontium americanum CA11
94
100
Parengyodontium americanum AZ2 ET
Parengyodontium americanum CA13
P. album subclade 3
Parengyodontium torokii LEC01
Parengyodontium torokii UAMH 9836
Parengyodontium torokii CBS 368.72
Parengyodontium torokii sp. nov. JC1038 ET
Engyodontium rectidentatum CBS 641.74
91
Engyodontium rectidentatum CBS 547.82
Engyodontium rectidentatum CBS 206.74
91
Engyodontium parvisporum IHEM 22910
Lecanicillium tenuipes CBS 658.80
Isaria coleopterora CBS 110.73

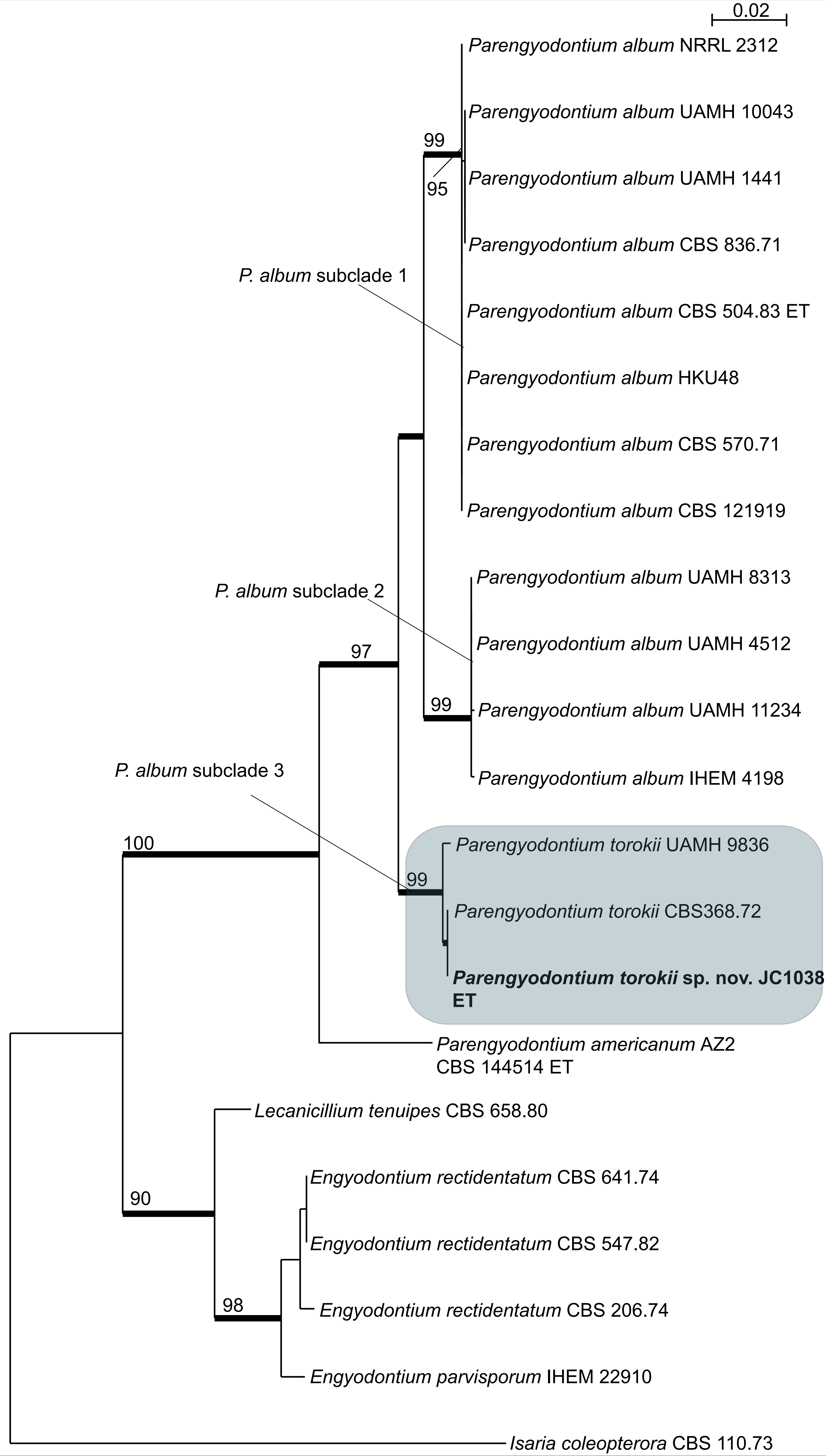
0.02
Parengyodontium album NRRL 2312
Parengyodontium album UAMH 10043
Parengyodontium album UAMH 1441
Parengyodontium album CBS 836.71
Parengyodontium album CBS 504.83 ET
Parengyodontium album HKU48
Parengyodontium album CBS 570.71
Parengyodontium album CBS 121919
Parengyodontium album UAMH 8313
Parengyodontium album UAMH 4512
Parengyodontium album UAMH 11234
Parengyodontium album IHEM 4198
Parengyodontium torokii UAMH 9836
Parengyodontium torokii CBS368.72
Parengyodontium torokii sp. nov. JC1038 ET
Parengyodontium americanum AZ2 CBS 144514 ET
Lecanicillium tenuipes CBS 658.80
Engyodontium rectidentatum CBS 641.74
Engyodontium rectidentatum CBS 547.82
Engyodontium rectidentatum CBS 206.74
Engyodontium parvisporum IHEM 22910
Isaria coleopterora CBS 110.73
P. album subclade 1
P. album subclade 2
P. album subclade 3
99
95
97
99
100
99
90
98

**Figure S4. Biofilm structures of *P. torokii* FJII-L10-SW-P1 strain.** **(A)** A thick layer of biofilm mat containing conidial spores and fungal mycelia. White arrow is pointing the sheath of fungal biofilm mat. **(B)** Arrow pointing to the white material where conidial spores are positioned. **(C)** Arrow pointing to a pair of conidia that were attached to the white fibrous material. **(D)** A thin sheath of fungal biofilm mat containing only conidia. Note only conidial spores are associated with these structures and not fungal mycelia

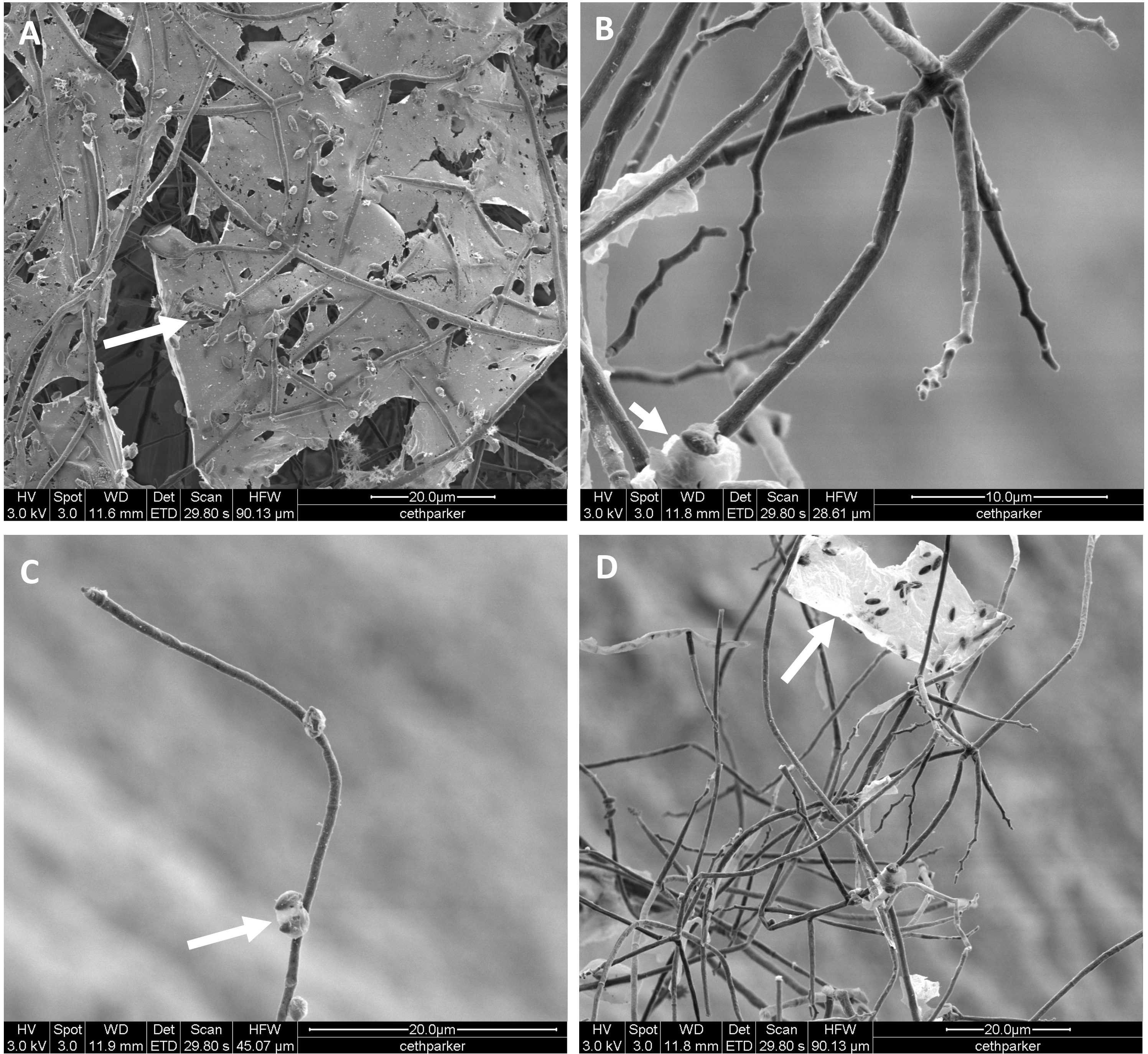

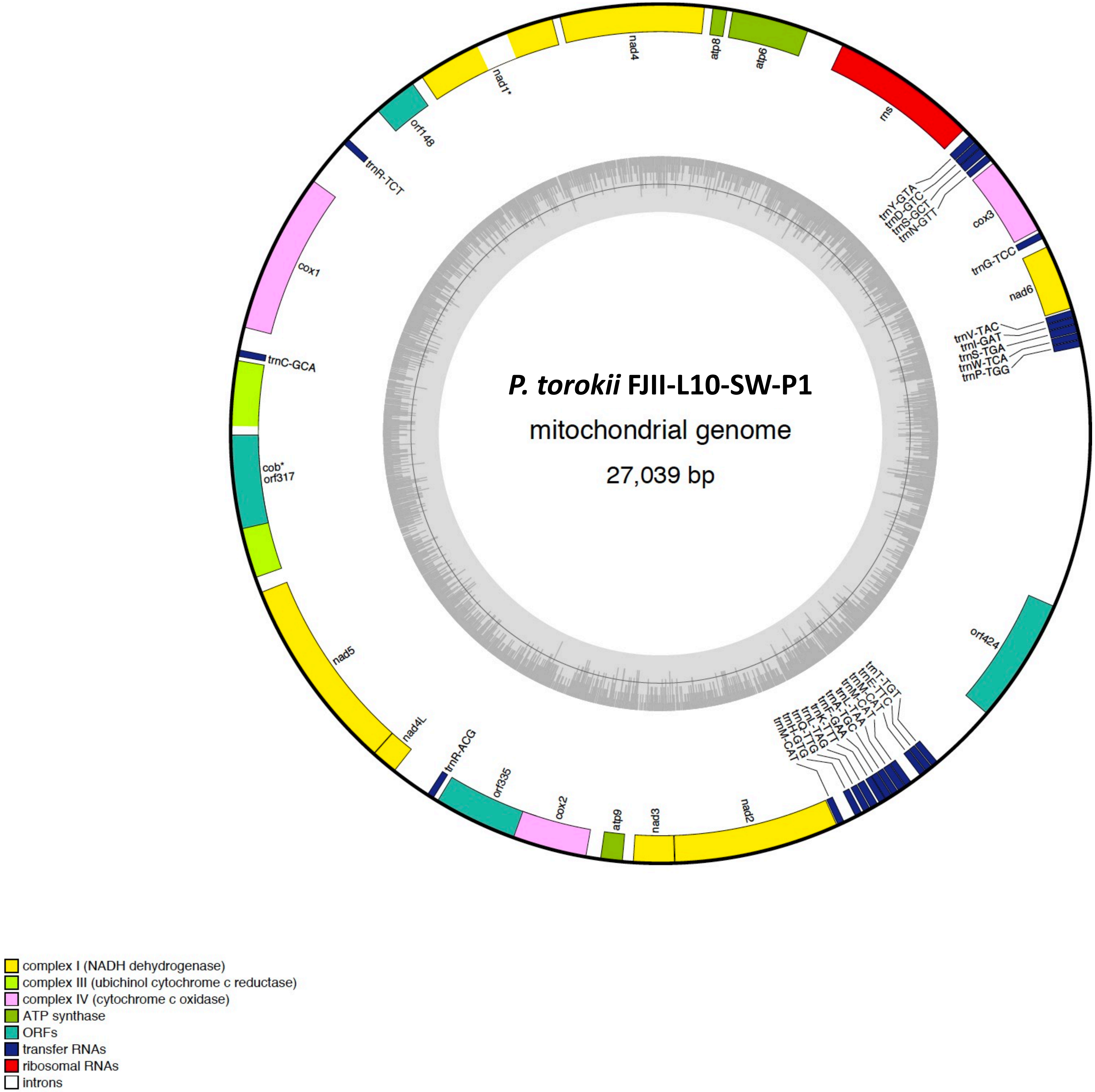

*P. torokii* FJII-L10-SW-P1
mitochondrial genome
27,039 bp
nad4
atp8
atp9
rns
trnY-GTA
trnD-GTC
trnS-GCT
trnN-GTT
cox3
trnG-TCC
nad6
trnV-TAC
trnI-GAT
trnS-TGA
trnW-TCA
trnP-TGG
orf424
trnT-TGT
trnE-TTC
trnM-CAT
trnM-CAT
trnL-TAA
trnA-TGC
trnF-GAA
trnK-TTT
trnL-TAG
trnQ-TTG
trnH-GTG
trnM-CAT
nad2
nad3
atp9
cox2
orf335
trnR-ACG
nad4L
nad5
cob*
orf317
trnC-GCA
cox1
trnR-TCT
orf148
nad1*
complex I (NADH dehydrogenase)
complex III (ubichinol cytochrome c reductase)
complex IV (cytochrome c oxidase)
ATP synthase
ORFs
transfer RNAs
ribosomal RNAs
introns

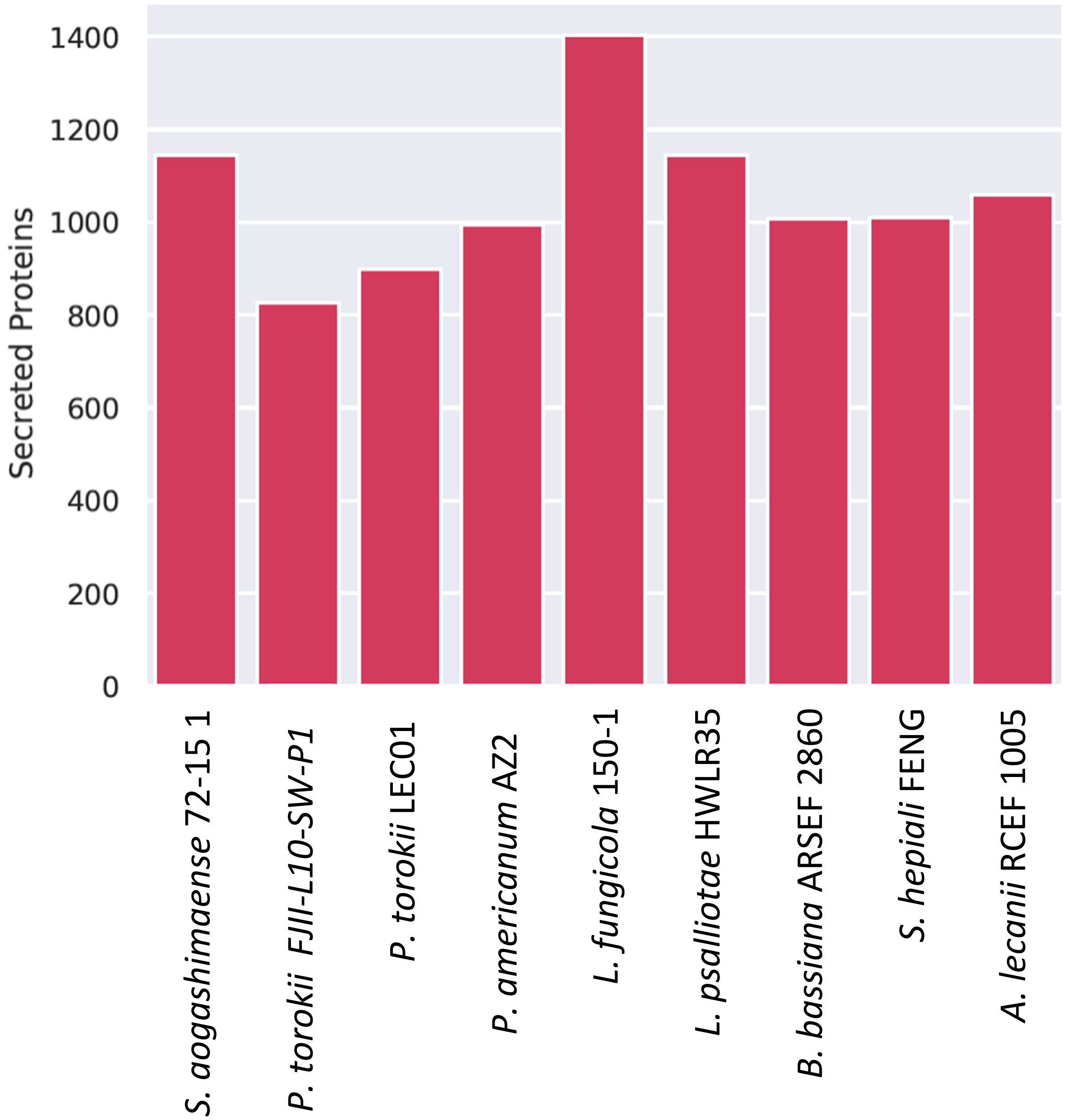

Secreted Proteins
1400
1200
1000
800
600
400
200
0
S. aogashimaense 72-15 1
P. torokii FJII-L10-SW-P1
P. torokii LEC01
P. americanum AZ2
L. fungicola 150-1
L. psalliotae HWLR35
B. bassiana ARSEF 2860
S. hepiali FENG
A. lecanii RCEF 1005

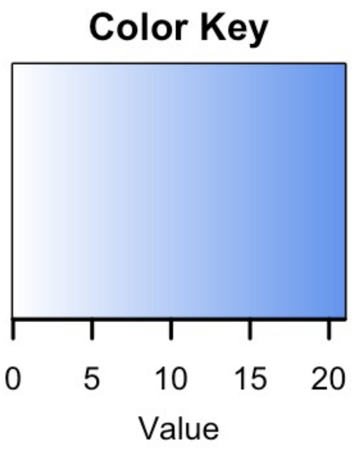


| | *S. aogashimaense* 72-15 1 | *P. torokii* FJII-L10-SW-P1 | *P. toroki* LEC01 | *P. americanum* AZ2 | *L. fungicola* 150-1 | *L. psalliotae* HWLR35 | *B. bassiana* ARSEF 2860 | *S. hepiali* FENG | *A. lecanii* RCEF 1005 |
|---|---|---|---|---|---|---|---|---|---|
| NAPAA | 1 | 1 | 1 | 0 | 2 | 1 | 1 | 1 | 1 |
| NRPS | 11 | 9 | 9 | 4 | 17 | 7 | 14 | 12 | 14 |
| NRPS-like | 10 | 4 | 4 | 4 | 10 | 6 | 4 | 8 | 5 |
| NRPS,terpene | 0 | 0 | 0 | 0 | 0 | 0 | 0 | 0 | 2 |
| NRPS-like,terpene | 0 | 0 | 1 | 0 | 0 | 0 | 0 | 0 | 0 |
| NRPS-like,T1PKS | 1 | 2 | 0 | 0 | 0 | 0 | 0 | 0 | 0 |
| NRPS,NRPS-like | 0 | 1 | 1 | 0 | 0 | 1 | 0 | 0 | 0 |
| NRPS,T1PKS | 2 | 2 | 4 | 1 | 3 | 3 | 3 | 4 | 2 |
| NRPS,betalactone | 1 | 0 | 0 | 0 | 1 | 0 | 0 | 0 | 0 |
| PKS | 0 | 0 | 0 | 0 | 0 | 1 | 0 | 0 | 0 |
| T1PKS | 11 | 7 | 9 | 6 | 21 | 10 | 10 | 10 | 5 |
| T1PKS,NRPS | 1 | 2 | 1 | 3 | 3 | 1 | 1 | 5 | 1 |
| T1PKS,NRPS-like | 1 | 1 | 3 | 0 | 0 | 0 | 0 | 1 | 0 |
| T3PKS | 0 | 0 | 0 | 0 | 0 | 0 | 0 | 1 | 0 |
| T3PKS,T1PKS | 0 | 0 | 0 | 0 | 0 | 0 | 1 | 0 | 0 |
| terpene | 4 | 3 | 3 | 3 | 11 | 5 | 7 | 3 | 4 |
| terpene,NRPS | 0 | 0 | 0 | 0 | 0 | 0 | 1 | 0 | 0 |
| phosphonate | 0 | 0 | 0 | 1 | 0 | 0 | 0 | 0 | 0 |
| siderophore | 0 | 0 | 0 | 1 | 0 | 0 | 0 | 0 | 0 |
| betalactone | 1 | 0 | 0 | 0 | 0 | 0 | 0 | 0 | 0 |
| fungal-RiPP,terpene | 1 | 0 | 0 | 0 | 0 | 0 | 0 | 0 | 0 |
| indole | 1 | 0 | 0 | 0 | 2 | 0 | 0 | 1 | 0 |

**Supplemental Table Captions.**

**Table S1.** Interspersed repeats and low complexity DNA sequences deduced by RepeatMasker in the *Parengyodontium torokii* FJII-L10-SW-P1 genome**.**

**Table S2.** Carbohydrate-active enzymes (CAZymes) profile of nine Cordycipitaceae fungal species

**Table S3.** Genes related to formation of biofilm, adherence, and glycosaminoglycan (GAG) biosynthesis characterized in *Aspergillus fumigatus* and identified in the *Parengyodontium torokii* genome.

**Table S4.** Genes involved in melanin biosynthesis, radioresistance and microgravity resistance and identified in the *Parengyodontium torokii* FJII-L10-SW-P1 genome.

**Table S5.** Secondary metabolite clusters identified by antiSMASH v6. in the *Parengyodontium torokii* FJII-L10-SW-P1 genome. The genomic scaffold, type, position, most similar known cluster and similarity are listed in the table.

**Table S6.** LC-MS analysis of *Parengyodontium torokii* FJ11-L10-SW-P1 based on identified biosynthetic gene clusters. Dereplication results of FJ11-L10-SW-P1 grown on rice media compared to an in-house database. Dereplication results of FJ11-L10-SW-P1 grown on oatmeal media compared to in-house database

Table S1 - Genomes download from NCBI and used for comparative genomic analyses with the novel Parengyodontium torokii strain FJII-L10-SW-P1

| Species | Strain | accession | Reference (PMID) |
|---|---|---|---|
| *Simplicillium aogashimaense* | 72-15.1 | JAALXG000000000.1 | 32616631 |
| *Lecanicillium sp.* | LEC01 | NIWZ00000000.2 | 30975820 |
| *Parengyodontium americanum* | AZ2 | PXYO00000000.1 | 32028048 |
| *Lecanicillium fungicola* | 150-1 | FWCC00000000.1 | 31072880 |
| *Lecanicillium psalliotae* | HWLR35 | PHFE00000000.1 | 29326219 |
| *Beauveria bassiana* | ARSEF 2860 | ADAH00000000.1 | 22761991 |
| *Samsoniella hepiali* | FENG | LNDK00000000.2 | Unpublished |
| *Akanthomyces lecanii* | RCEF 1005 | AZHF00000000.1 | 27071652 |

**Supplemental Table S2: Interspersed repeats and low complexity DNA sequences deduced by RepeatMasker in the *Parengyodontium torokii* FJII-L10-SW-P1 genome**

| Characteristics | number of elements | length occupied (bp) | percentage of the genome (%) |
|---|---|---|---|
| **Retroelements** | 257 | 69,759 | 0.23 |
| **Penelope** | 2 | 115 | 0 |
| **LINEs** | 37 | 7,368 | 0.02 |
| **CRE/SLACS** | 1 | 453 | 0 |
| **R1/LOA/Jockey** | 2 | 271 | 0 |
| **LTR elements** | 220 | 62,391 | 0.21 |
| **Ty1/Copia** | 49 | 5,535 | 0.02 |
| **Gypsy/DIRS1** | 122 | 45,845 | 0.15 |
| | | | |
| **DNA transposons** | 185 | 89,975 | 0.3 |
| **hobo-Activator** | 16 | 4,741 | 0.02 |
| **Tc1-IS630-Pogo** | 58 | 20,832 | 0.07 |
| **En-Spm** | 0 | 0 | 0 |
| **MuDR-IS905** | 0 | 0 | 0 |
| **PiggyBac** | 17 | 15,249 | 0.05 |
| **Tourist/Harbinger** | 4 | 259 | 0 |
| **Other (Mirage, P-element, Transib)** | 1 | 85 | 0 |
| | | | |
| **Rolling-circles** | 1 | 70 | 0 |
| **Unclassified** | 220 | 49,926 | 0.16 |
| **Small RNA** | 7 | 5,524 | 0.02 |
| | | | |
| **Simple repeats** | 6431 | 239,287 | 0.79 |
| **Low complexity** | 728 | 33,542 | 0.11 |

**Supplemental Table S3. Full Carbohydrate-active enzymes (CAZymes) profile of 9 Cordycipitaceae fungal species**

| | S. aogashimaense 72-15_1 | P. toroki JC1038 | L. sp. LEC01 | Parengyodontium_americanum | L. fungicola 150-1 | Lecanicillium_psalliotae_HWLR35 | B. bassiana ARSEF_2860 | S. hepiali FENG | A. lecanii RCEF_1005 |
|---|---|---|---|---|---|---|---|---|---|
| AA1 | 7 | 8 | 9 | 9 | 8 | 7 | 11 | 9 | 6 |
| AA11 | 3 | 4 | 4 | 5 | 6 | 5 | 4 | 6 | 6 |
| AA12 | 2 | 2 | 2 | 2 | 3 | 3 | 2 | 2 | 3 |
| AA13 | 1 | 0 | 0 | 0 | 0 | 0 | 0 | 0 | 0 |
| AA14 | 1 | 1 | 1 | 1 | 3 | 1 | 0 | 1 | 1 |
| AA2 | 6 | 2 | 2 | 3 | 2 | 5 | 3 | 3 | 4 |
| AA3 | 25 | 15 | 15 | 15 | 36 | 15 | 13 | 12 | 13 |
| AA4 | 2 | 1 | 1 | 1 | 1 | 1 | 2 | 0 | 0 |
| AA5 | 1 | 2 | 1 | 0 | 4 | 2 | 3 | 2 | 2 |
| AA6 | 1 | 0 | 1 | 1 | 4 | 1 | 1 | 1 | 1 |
| AA7 | 19 | 12 | 11 | 13 | 19 | 12 | 16 | 10 | 13 |
| AA8 | 4 | 4 | 3 | 2 | 4 | 2 | 2 | 2 | 2 |
| AA9 | 2 | 1 | 1 | 1 | 1 | 1 | 1 | 1 | 1 |
| CBM13 | 0 | 0 | 0 | 1 | 1 | 0 | 0 | 0 | 1 |
| CBM18 | 0 | 0 | 0 | 0 | 0 | 0 | 0 | 1 | 0 |
| CBM20 | 1 | 2 | 2 | 2 | 1 | 0 | 1 | 1 | 0 |
| CBM21 | 1 | 1 | 1 | 1 | 1 | 1 | 1 | 1 | 1 |
| CBM24 | 1 | 1 | 1 | 1 | 2 | 1 | 0 | 0 | 0 |
| CBM32 | 1 | 1 | 1 | 0 | 2 | 1 | 2 | 1 | 1 |
| CBM38 | 1 | 0 | 0 | 0 | 0 | 1 | 0 | 0 | 0 |
| CBM39 | 0 | 0 | 0 | 0 | 1 | 0 | 0 | 0 | 0 |
| CBM42 | 3 | 1 | 1 | 3 | 1 | 4 | 1 | 3 | 4 |
| CBM43 | 1 | 1 | 1 | 2 | 1 | 0 | 0 | 0 | 0 |
| CBM50 | 2 | 2 | 3 | 6 | 1 | 2 | 3 | 2 | 4 |
| CBM52 | 0 | 0 | 0 | 0 | 0 | 1 | 0 | 0 | 0 |
| CBM66 | 1 | 1 | 2 | 1 | 5 | 6 | 1 | 3 | 3 |
| CBM67 | 1 | 0 | 0 | 2 | 1 | 1 | 0 | 1 | 1 |
| CE1 | 4 | 3 | 3 | 3 | 4 | 2 | 2 | 2 | 2 |
| CE10 | 31 | 24 | 27 | 29 | 35 | 19 | 20 | 22 | 23 |
| CE16 | 4 | 1 | 2 | 2 | 1 | 2 | 1 | 1 | 1 |
| CE3 | 6 | 3 | 2 | 5 | 2 | 4 | 1 | 3 | 4 |
| CE4 | 3 | 4 | 4 | 4 | 4 | 3 | 3 | 3 | 3 |
| CE5 | 8 | 5 | 3 | 2 | 5 | 10 | 5 | 8 | 14 |
| CE9 | 1 | 1 | 1 | 1 | 1 | 1 | 1 | 1 | 1 |
| GH1 | 1 | 3 | 3 | 3 | 2 | 1 | 1 | 1 | 2 |
| GH10 | 0 | 0 | 0 | 0 | 0 | 0 | 1 | 0 | 0 |
| GH105 | 4 | 2 | 2 | 2 | 9 | 2 | 0 | 1 | 2 |
| GH109 | 1 | 1 | 1 | 1 | 0 | 0 | 0 | 0 | 1 |

| | S. aogashimaense 72-15_1 | P. toroki JC1038 | L. sp. LEC01 | Parengyodontium_americanum | L. fungicola 150-1 | Lecanicillium_psalliotae_HWLR35 | B. bassiana ARSEF_2860 | S. hepiali FENG | A. lecanii RCEF_1005 |
|---|---|---|---|---|---|---|---|---|---|
| GH11 | 1 | 1 | 1 | 0 | 0 | 0 | 0 | 0 | 0 |
| GH114 | 2 | 2 | 2 | 2 | 1 | 3 | 1 | 1 | 1 |
| GH115 | 0 | 1 | 1 | 1 | 0 | 0 | 0 | 0 | 0 |
| GH12 | 2 | 1 | 1 | 1 | 2 | 0 | 1 | 1 | 1 |
| GH125 | 3 | 3 | 3 | 3 | 2 | 3 | 3 | 3 | 3 |
| GH127 | 0 | 0 | 0 | 0 | 1 | 0 | 0 | 0 | 1 |
| GH128 | 4 | 3 | 3 | 3 | 5 | 9 | 3 | 7 | 7 |
| GH13 | 8 | 8 | 8 | 7 | 8 | 5 | 4 | 5 | 5 |
| GH131 | 0 | 0 | 0 | 0 | 0 | 1 | 0 | 0 | 0 |
| GH132 | 2 | 2 | 2 | 3 | 5 | 2 | 2 | 2 | 2 |
| GH133 | 1 | 1 | 1 | 1 | 1 | 1 | 1 | 1 | 1 |
| GH134 | 1 | 0 | 0 | 0 | 0 | 0 | 0 | 0 | 0 |
| GH135 | 1 | 0 | 0 | 0 | 1 | 1 | 0 | 1 | 0 |
| GH136 | 1 | 1 | 1 | 0 | 1 | 2 | 1 | 1 | 1 |
| GH141 | 0 | 0 | 1 | 0 | 0 | 0 | 0 | 0 | 0 |
| GH142 | 1 | 1 | 1 | 1 | 1 | 1 | 1 | 1 | 1 |
| GH15 | 1 | 3 | 3 | 2 | 2 | 2 | 2 | 2 | 2 |
| GH152 | 0 | 0 | 1 | 0 | 1 | 1 | 0 | 0 | 0 |
| GH154 | 2 | 1 | 1 | 0 | 5 | 1 | 1 | 1 | 1 |
| GH16 | 18 | 16 | 17 | 16 | 19 | 20 | 20 | 22 | 20 |
| GH162 | 1 | 0 | 0 | 1 | 1 | 0 | 1 | 1 | 1 |
| GH17 | 4 | 2 | 2 | 2 | 2 | 2 | 2 | 2 | 2 |
| GH18 | 28 | 15 | 19 | 21 | 31 | 26 | 19 | 26 | 25 |
| GH2 | 11 | 9 | 10 | 8 | 10 | 8 | 7 | 8 | 8 |
| GH20 | 6 | 8 | 8 | 8 | 6 | 8 | 4 | 6 | 7 |
| GH24 | 4 | 2 | 2 | 4 | 1 | 2 | 0 | 0 | 0 |
| GH25 | 1 | 1 | 1 | 2 | 2 | 2 | 1 | 2 | 1 |
| GH27 | 0 | 0 | 0 | 0 | 2 | 0 | 0 | 0 | 0 |
| GH28 | 2 | 1 | 1 | 1 | 2 | 1 | 1 | 1 | 1 |
| GH29 | 3 | 3 | 3 | 4 | 1 | 2 | 2 | 2 | 2 |
| GH3 | 11 | 13 | 12 | 13 | 15 | 14 | 11 | 10 | 12 |
| GH30 | 2 | 1 | 1 | 1 | 1 | 2 | 0 | 0 | 0 |
| GH31 | 7 | 5 | 6 | 6 | 8 | 7 | 6 | 5 | 7 |
| GH32 | 2 | 1 | 1 | 2 | 1 | 2 | 1 | 1 | 1 |
| GH33 | 0 | 2 | 2 | 3 | 0 | 0 | 0 | 0 | 0 |
| GH35 | 2 | 2 | 3 | 5 | 3 | 3 | 3 | 3 | 3 |
| GH36 | 2 | 2 | 2 | 3 | 3 | 2 | 1 | 2 | 2 |
| GH37 | 2 | 2 | 2 | 2 | 3 | 2 | 2 | 2 | 2 |
| GH38 | 1 | 1 | 1 | 1 | 3 | 1 | 1 | 1 | 1 |
| GH4 | 1 | 0 | 0 | 0 | 0 | 0 | 0 | 0 | 0 |
| GH43 | 6 | 4 | 4 | 4 | 1 | 5 | 2 | 3 | 3 |

| | S. aogashimaense 72-15_1 | P. toroki JC1038 | L. sp. LEC01 | Parengyodontium_americanum | L. fungicola 150-1 | Lecanicillium_psalliotae_HWLR35 | B. bassiana ARSEF_2860 | S. hepiali FENG | A. lecanii RCEF_1005 |
|---|---|---|---|---|---|---|---|---|---|
| GH45 | 1 | 0 | 0 | 0 | 0 | 1 | 0 | 0 | 1 |
| GH47 | 8 | 9 | 9 | 9 | 7 | 8 | 7 | 8 | 8 |
| GH5 | 12 | 9 | 9 | 7 | 13 | 8 | 7 | 8 | 9 |
| GH51 | 0 | 0 | 1 | 1 | 0 | 0 | 0 | 0 | 0 |
| GH53 | 0 | 0 | 1 | 0 | 0 | 0 | 0 | 0 | 0 |
| GH54 | 3 | 1 | 1 | 4 | 1 | 4 | 1 | 3 | 4 |
| GH55 | 7 | 3 | 3 | 5 | 14 | 9 | 8 | 10 | 4 |
| GH6 | 1 | 0 | 0 | 0 | 0 | 0 | 0 | 0 | 0 |
| GH64 | 4 | 2 | 2 | 2 | 2 | 2 | 1 | 2 | 2 |
| GH65 | 2 | 1 | 1 | 1 | 1 | 1 | 0 | 1 | 1 |
| GH67 | 0 | 0 | 0 | 1 | 0 | 0 | 0 | 0 | 0 |
| GH7 | 1 | 0 | 0 | 0 | 0 | 0 | 0 | 0 | 0 |
| GH71 | 3 | 1 | 1 | 2 | 3 | 1 | 0 | 1 | 2 |
| GH72 | 5 | 6 | 6 | 6 | 6 | 5 | 6 | 5 | 5 |
| GH75 | 5 | 4 | 4 | 4 | 25 | 8 | 2 | 5 | 5 |
| GH76 | 19 | 11 | 12 | 14 | 9 | 11 | 8 | 11 | 11 |
| GH78 | 2 | 1 | 1 | 3 | 2 | 3 | 0 | 2 | 2 |
| GH79 | 5 | 1 | 1 | 3 | 3 | 4 | 2 | 4 | 5 |
| GH81 | 2 | 2 | 2 | 2 | 2 | 2 | 1 | 2 | 2 |
| GH84 | 1 | 1 | 1 | 1 | 1 | 1 | 1 | 1 | 1 |
| GH88 | 3 | 3 | 3 | 1 | 7 | 2 | 1 | 1 | 2 |
| GH89 | 3 | 3 | 2 | 2 | 1 | 2 | 2 | 3 | 2 |
| GH92 | 4 | 5 | 5 | 5 | 5 | 5 | 4 | 4 | 4 |
| GH93 | 2 | 2 | 3 | 1 | 0 | 0 | 0 | 1 | 1 |
| GH95 | 2 | 1 | 1 | 1 | 4 | 1 | 1 | 1 | 1 |
| GT1 | 5 | 4 | 4 | 4 | 8 | 2 | 5 | 3 | 2 |
| GT15 | 4 | 4 | 4 | 4 | 3 | 3 | 3 | 3 | 3 |
| GT17 | 2 | 2 | 2 | 2 | 5 | 2 | 4 | 2 | 2 |
| GT2_Chitin_synth | 6 | 6 | 6 | 6 | 6 | 6 | 6 | 6 | 6 |
| GT2_Glyco_tranf_2 | 2 | 1 | 2 | 2 | 3 | 2 | 2 | 2 | 2 |
| GT2_Glyco_trans_2 | 5 | 2 | 2 | 2 | 0 | 2 | 3 | 2 | 2 |
| GT2_Glycos_transf | 2 | 2 | 2 | 2 | 2 | 2 | 2 | 2 | 2 |
| GT20 | 4 | 7 | 7 | 4 | 4 | 4 | 4 | 6 | 4 |
| GT21 | 1 | 1 | 1 | 1 | 1 | 1 | 1 | 1 | 1 |
| GT22 | 4 | 4 | 4 | 4 | 4 | 4 | 4 | 4 | 4 |
| GT24 | 1 | 0 | 0 | 1 | 1 | 1 | 1 | 1 | 0 |
| GT25 | 1 | 2 | 1 | 5 | 7 | 5 | 5 | 5 | 4 |
| GT3 | 1 | 1 | 1 | 1 | 1 | 1 | 1 | 1 | 1 |
| GT31 | 0 | 0 | 0 | 0 | 0 | 1 | 0 | 0 | 0 |
| GT32 | 4 | 4 | 4 | 5 | 3 | 3 | 4 | 3 | 3 |
| GT33 | 1 | 1 | 1 | 1 | 0 | 1 | 1 | 1 | 1 |

| | S. aogashimaense 72-15_1 | P. toroki JC1038 | L. sp. LEC01 | Parengyodontium_americanum | L. fungicola 150-1 | Lecanicillium_psalliotae_HWLR35 | B. bassiana ARSEF_2860 | S. hepiali FENG | A. lecanii RCEF_1005 |
|---|---|---|---|---|---|---|---|---|---|
| GT34 | 2 | 4 | 5 | 8 | 2 | 4 | 2 | 2 | 3 |
| GT35 | 1 | 1 | 1 | 1 | 1 | 1 | 1 | 1 | 1 |
| GT39 | 3 | 3 | 3 | 3 | 3 | 3 | 3 | 3 | 3 |
| GT4 | 4 | 4 | 4 | 4 | 4 | 4 | 4 | 4 | 5 |
| GT48 | 1 | 2 | 2 | 2 | 2 | 2 | 1 | 2 | 2 |
| GT50 | 1 | 1 | 1 | 1 | 0 | 1 | 1 | 1 | 1 |
| GT57 | 2 | 2 | 2 | 2 | 2 | 2 | 2 | 2 | 2 |
| GT58 | 1 | 1 | 1 | 1 | 1 | 1 | 1 | 1 | 1 |
| GT59 | 1 | 0 | 0 | 1 | 1 | 1 | 1 | 1 | 1 |
| GT62 | 3 | 3 | 3 | 3 | 3 | 3 | 3 | 3 | 3 |
| GT64 | 1 | 0 | 0 | 1 | 0 | 1 | 2 | 1 | 0 |
| GT66 | 1 | 1 | 1 | 1 | 1 | 1 | 1 | 1 | 1 |
| GT69 | 5 | 3 | 4 | 2 | 3 | 4 | 3 | 3 | 4 |
| GT71 | 0 | 0 | 0 | 0 | 1 | 1 | 1 | 1 | 1 |
| GT76 | 1 | 1 | 1 | 1 | 1 | 1 | 1 | 1 | 1 |
| GT8 | 2 | 2 | 2 | 2 | 2 | 2 | 2 | 2 | 2 |
| GT90 | 6 | 3 | 3 | 4 | 3 | 1 | 3 | 1 | 2 |
| PL1 | 1 | 0 | 0 | 0 | 0 | 0 | 0 | 0 | 0 |
| PL20 | 1 | 1 | 1 | 1 | 1 | 1 | 0 | 1 | 1 |
| PL3 | 1 | 0 | 0 | 0 | 0 | 0 | 0 | 0 | 0 |
| PL35 | 0 | 0 | 0 | 0 | 1 | 0 | 0 | 0 | 0 |
| PL7 | 4 | 0 | 0 | 2 | 17 | 4 | 1 | 1 | 1 |
| PL8 | 1 | 1 | 1 | 1 | 1 | 1 | 1 | 1 | 1 |

Supplemental Table S4. Genes related to formation of biofilm, adherence and glycosaminoglycan (GAG) biosynthesis characterized in *Aspergillus fumigatus* and identified in the *Pare*

| gene name in *A. fumigatus* | gene ID in *A. fumigatus* | Predicted function | Best match in P. torokii |
|---|---|---|---|
| uge3 | Afu3g07910 | UDP-glucose epimerase required for GAG synthesis. Null mutants are unable to adhere to abiotic surfaces and exhibit reduced adherence to epithelial cells | H0188_002713 |
| medA | Afu2g13260 | Transcription factor that regulates GAG biosynthetic cluster. Null mutants have reduced *uge3* expression and reduced adhesion | H0188_004544 |
| stuA | Afu2g07900 | Transcription factor that regulates GAG biosynthetic cluster. Null mutants have reduced *uge3* expression and reduced adhesion | H0188_006447 |
| somA | Afu7g02260 | Transcription factor, upstream regulator of *medA* and *stuA*. Role in GAG biosynthesis regulation. Null mutants have decreased adherence | H0188_006643 |
| mpkA | Afu4g13720 | MAP kinase, null mutants have decreased adherence, cell wall integrity | **H0188_008493** |
| sitA | Afu6g11470 | MpkA-regulating phosphatase, upstream regulator of *mpkA* in coordinating surface adhesion and cell wall composition | H0188_008416 |
| gtb3 | Afu3g07860 | Has domain(s) with predicted role in biosynthetic process | X |
| ega3 | AFUA_3G07890 | endo-α-1,4-polygalactosaminidase | H0188_003078 |
| sph3 | Afu3g07900 | α-1,4-galactosaminogalactan hydrolase | X |
| agd3 | Afu3g07870 | Extracellular serine rich protein; predicted secretory signal sequence; ortholog of *A. nidulans* AN2954 | H0188_008471 |
| - | Afu2g01700 | AMP-activated protein kinase activity | H0188_000928 |
| exg1 | Afu1g03600 | glucan endo-1,6-beta-glucosidase activity | H0188_003673 |
| bgt1 | Afu1g11460 | 1,3-beta-glucanosyltransferase | H0188_003464 |
| adh1 | Afu7g01010 | alcohol dehydrogenase | H0188_004408 |
| acyA | Afu6g08520 | Adenylate cyclase of the cAMP-dependent signaling pathway | H0188_005876 |
| rhbA | Afu5g05480 | Ras-related signaling protein | H0188_008959 |
| rasA | Afu5g11230 | Ras family GTPase protein | H0188_002821 |
| pkaC | Afu2g12200 | cAMP-dependent protein kinase catalytic subunit | H0188_008806 |
| mpkA | Afu4g13720 | Mitogen-activated protein kinase | H0188_008493 |
| - | Afu6g11010 | Ortholog(s) have role in budding cell apical bud growth, cell budding, cell morphogenesis, cellular response to biotic stimulus, cellular response to drug and cellular response to neutral pH | H0188_003706 |
| - | Afu3g14040 | Ortholog(s) have role in filamentous growth of a population of unicellular organisms and growth of unicellular organism as a thread of attached cells | H0188_002587 |
| - | Afu3g08050 | Ortholog(s) have DNA-binding transcription factor activity, RNA polymerase II-specific, RNA polymerase II transcription regulator recruiting activity, transcription coactivator activity | H0188_004740 |
| - | Afu2g17860 | Ortholog(s) have role in cellular response to drug, growth of symbiont in host and positive regulation of cell adhesion involved in single-species biofilm formation | H0188_005262 |
| - | Afu2g10640 | Ortholog(s) have chromatin binding, histone acetyltransferase activity, phosphatidylserine binding, sequence-specific DNA binding, transcription coactivator activity, transcription regulator activity | H0188_009550 |
| - | Afu2g03460 | Ortholog(s) have DNA-binding transcription activator activity, RNA polymerase II-specific, DNA-binding transcription repressor activity, RNA polymerase II-specific activity | H0188_007512 |
| - | Afu1g17150 | Ortholog(s) have role in cellular carbohydrate metabolic process, maltose catabolic process, maltose metabolic process and positive regulation of cell adhesion involved in single-species biofilm formation | H0188_001623 |
| - | Afu1g01850 | Ortholog(s) have DNA-binding transcription factor activity, RNA polymerase II-specific activity and role in negative regulation of single-species biofilm formation, negative regulation of transcription by RNA polymerase II | H0188_000557 |
| - | Afu3g13790 | Ortholog(s) have calcium transmembrane transporter activity, phosphorylative mechanism, ion transmembrane transporter activity, phosphorylative mechanism activity | H0188_004626 |
| zfpB | Afu1g10230 | Putative C2H2 zinc-finger transcription factor | H0188_005857 |
| scw4 | Afu6g12380 | Ortholog(s) have role in cell adhesion, cell adhesion involved in single-species biofilm formation, cell-substrate adhesion, cellular response to glucose starvation and cellular response to neutral pH, | H0188_003464 |
| mnt1 | Afu5g10760 | Putative alpha-1,2-mannosyltransferase with a predicted role in protein glycosylation; 1 predicted transmembrane domain | H0188_002879 |
| - | Afu4g04070 | Protein of unknown function, phoB-regulated | H0188_005032 |
| sitA | Afu6g11470 | Serine/threonine phosphatase required for cell wall integrity, biofilm formation, adhesion and virulence | H0188_008416 |
| - | Afu4g13660 | Ortholog(s) have efflux transmembrane transporter activity, polyamine transmembrane transporter activity | H0188_007721 |
| - | Afu3g03190 | Ortholog(s) have cation transmembrane transporter activity, efflux transmembrane transporter activity, xenobiotic transmembrane transporter activity (1) | H0188_003803 |
| yvc1 | Afu3g13490 | Calcium activated cation channel with a predicted role in cellular cation homeostasis | H0188_004780 |
| ptcB | Afu1g09280 | Putative type 2C protein phosphatase (PP2C) involved in dephosphorylation of SakA MAP kinase in response to osmotic stress | H0188_004047 |

Supplemental Table S5 - Genes involved in melanin biosynthesis, radioresistance and microgravity resistance.

| | | |
|---|---|---|
| **Melanin biosynthesis** | | |
| **DHN-melanin pathway** | | |
| Gene ID in *Aspergillus fumigatus* | Gene ID in *Aspergillus niger* | Homologue in *Parengyodontium torokii* |
| pksP/Afu2g17600 | An03g05440 | H0188_000958 |
| ayg1/Afu2g17550 | An14g05350 | H0188_005280 |
| arp2/Afu2g17560 | An02g00220 | H0188_009464 |
| arp1/Afu2g17580 | An08g09920 | X |
| abr2/Afu2g17530 | An01g1366 | H0188_000003 |
| abr1/Afu2g17540 | An14g05370 | H0188_005715 |
| **DOPA-melanin pathway** | | |
| melC2/Afu3g01070 | An01g09220 | H0188_002942 |
| melO/Afu1g17430 | An12g01670 | H0188_005733 |
| tpcJ/Afu4g14490 | An12g05810 | H0188_002651 |
| **L-Tyrosine degradation pathway** | | |
| tat/Afu2g13630 | An02g05540 | H0188_009650 |
| hppD/Afu2g04200 | An11g02200 | H0188_004175 |
| hmgA/Afu2g04220 | An11g02180 | H0188_000361 |
| fahA/Afu2g04230 | An11g02170 | H0188_000334 |
| maiA/Afu2g04240 | An11g02160 | H0188_008130 |
| | | |
| **Radioresistace genes** | | |
| rad51/Afu1g10410 | | H0188_003614 |
| rad54/Afu6g12910 | | H0188_003451 |
| rad54b/Afu1g15120 | | H0188_004866 |
| rdh54/Afu1g15120 | | H0188_003451 |
| rad9/Afu5g04020 | | H0188_001584 |
| rad9b/Afu2g10710 | | H0188_005646 |
| | | |
| **Microgravity resistance gene*** | | |
| Proteinase K (Parengyodontium album) - X14689.1 | | H0188_006801/H0188_008610 |
| Floculin A (Saccharomyces pastorianus) - 4LHK_A | | X |
| Floculin B (Saccharomyces pastorianus) - 4LHK_B | | X |
| DNA repair protein rad9 (Schizosaccharomyces pombe) - P26306 | | H0188_005646 |
| Daughter-specific expression protein 2 (Saccharomyces cerevisiae) - P38844.1 | | H0188_008471 |
| Daughter-specific expression protein 1 (Saccharomyces cerevisiae) - P40077 | | X |

* Query genes were selected from multiplefungal species

Table S6. Secondary metabolite clusters identified by antiSMASH v6. in the Parengyodontium torokii FJII-L10-SW-P1 genome.

| **Region (genomic Scaffold)** | **Type** | **From** | **To** | **Most similar known cluster** | | **Similarity** |
|---|---|---|---|---|---|---|
| Region 1.1 | NRPS | 235,392 | 283,546 | | | |
| Region 1.2 | NRPS-like | 499,503 | 542,84 | | | |
| Region 1.3 | T1PKS | 545,554 | 593,164 | | | |
| Region 2.1 | NRPS | 504,787 | 537,693 | | | |
| Region 7.1 | NRPS-like,T1PKS | 288 | 35,258 | | | |
| Region 7.2 | T1PKS,NRPS | 211,319 | 263,478 | equisetin | NRP + Polyketide | 45% |
| Region 8.1 | NRPS-like | 14,078 | 57,416 | | | |
| Region 9.1 | terpene | 186,065 | 207,624 | | | |
| Region 13.1 | NAPAA | 185,108 | 218,998 | | | |
| Region 14.1 | NRPS-like | 79,895 | 123,667 | | | |
| Region 19.1 | NRPS | 74,496 | 126,369 | | | |
| Region 21.1 | T1PKS | 155,29 | 201,697 | | | |
| Region 24.1 | T1PKS | 126,575 | 173,852 | | | |
| Region 25.1 | T1PKS | 169,887 | 206,955 | | | |
| Region 37.1 | NRPS,NRPS-like | 66,329 | 160,996 | cephalosporin C | NRP | 71% |
| Region 45.1 | T1PKS | 1 | 41,374 | | | |
| Region 46.1 | T1PKS,NRPS | 81,864 | 134,547 | | | |
| Region 49.1 | NRPS-like,T1PKS | 87,657 | 160,102 | EQ-4 | NRP | 100% |
| Region 70.1 | terpene | 86,187 | 107,762 | squalestatin S1 | Terpene | 40% |
| Region 76.1 | NRPS | 13,728 | 64,552 | curvupallide-B | NRP + Polyketide | 50% |
| Region 78.1 | NRPS,T1PKS | 1 | 41,975 | cytochalasin E / cytochalasin K | NRP + Polyketide:Iterative type I | 15% |
| Region 98.1 | NRPS | 16,327 | 63,391 | | | |
| Region 103.1 | NRPS | 1 | 45,747 | | | |
| Region 108.1 | NRPS,T1PKS | 9,595 | 61,452 | pyranonigrin E | Polyketide | 100% |
| Region 109.1 | NRPS-like | 27,908 | 71,988 | | | |
| Region 111.1 | T1PKS | 61,274 | 92,081 | | | |
| Region 132.1 | terpene | 39,107 | 60,084 | | | |
| Region 135.1 | T1PKS,NRPS-like | 21,748 | 71,69 | | | |
| Region 161.1 | NRPS | 1 | 41,834 | | | |
| Region 208.1 | NRPS | 1 | 45,777 | | | |
| Region 213.1 | T1PKS | 1 | 37,438 | | | |
| Region 220.1 | NRPS | 11,41 | 42,442 | dimethylcoprogen | NRP | 100% |

Supplemental Table S7. LC-MS analysis of *Parengyodontium torokii* FJ11-L10-SW-P1 based on identified biosynthetic gene clusters.

| Name of Compound | Standard m/z | Standard RT | Experimental m/z | Experimental RT | Library fragmentation Data | | | | | | | | | | Experimental Fragmentation Data | | | | | | | | | |
|---|---|---|---|---|---|---|---|---|---|---|---|---|---|---|---|---|---|---|---|---|---|---|---|---|
| | | | | | Frg 1 | Frg 2 | Frg 3 | Frg 4 | Frg 5 | Frg 6 | Frg 7 | Frg 8 | Frg 9 | Frg 10 | Frg 1 | Frg 2 | Frg 3 | Frg 4 | Frg 5 | Frg 6 | Frg 7 | Frg 8 | Frg 9 | Frg 10 |
| **Rice** | | | | | | | | | | | | | | | | | | | | | | | | |
| 5,8-Epidioxyergosta-6,9(11),22-trien-3-ol | 427.32 | 7.9 | 427.3197 | 7.78 | 409 | 391 | 381 | 363 | 339 | 303 | 285 | 267 | 175 | 147 | 409 | 391 | 381 | 339 | 311 | 301 | 285 | 283 | 267 | 239 |
| (E)-2,3-dihydroxypropyl octadec-6-enoate | 357.2996 | 7.98 | 357.2994 | 7.95 | 339 | 283 | 265 | 247 | 177 | 163 | 151 | 149 | 135 | 121 | 339 | 283 | 265 | 247 | 191 | 165 | 151 | 149 | 135 | 121 |
| Betulinan C | 291.1018 | 5.64 | 291.1011 | 5.72 | 291 | 276 | 273 | 263 | 260 | 259 | 258 | 245 | 231 | 213 | 291 | 276 | 273 | 263 | 260 | 259 | 258 | 245 | 231 | 213 |
| 6,9-Octadecadienoic acid | 281.2476 | 7.25 | 281.2473 | 7.21 | 264 | 263 | 246 | 245 | 221 | 163 | 151 | 149 | 135 | 121 | 264 | 263 | 246 | 245 | 185 | 180 | 176 | 166 | 150 | 136 |
| Cyclo (L-Pro-L-Leu) | 211.1437 | 1.89 | 211.1436 | 1.96 | 211 | 194 | 183 | 155 | 138 | 127 | 86 | 70 | | | 194 | 183 | 167 | 166 | 155 | 138 | 127 | 86 | 70 | 69 |
| Ergosta-4,6,8(14),22-tetraen-3-ol, (3β,22E)- | 395.3303 | 8.82 | 395.3298 | 8.82 | 377 | 325 | 311 | 307 | 293 | 269 | 253 | 251 | | | 377 | 335 | 325 | 311 | 307 | 293 | 271 | 269 | 253 | 251 |
| Cyclo (Pro-Phe) | 245.1281 | 2.29 | 245.128 | 2.32 | 245 | 228 | 217 | 200 | 172 | 154 | 131 | 120 | 70 | | 227 | 217 | 213 | 204 | 200 | 181 | 146 | 120 | | |
| **Oatmeal** | | | | | | | | | | | | | | | | | | | | | | | | |
| 5,8-Epidioxyergosta-6,9(11),22-trien-3-ol | 427.32 | 7.9 | 427.3196 | 7.79 | 409 | 391 | 381 | 363 | 339 | 303 | 285 | 267 | 175 | 147 | 427 | 410 | 409 | 391 | 382 | 339 | 301 | 285 | 283 | 267 |
| Cephalochromin | 519.1267 | 5.45 | 519.127 | 5.47 | 519 | 501 | 477 | 476 | 473 | 459 | 260 | 245 | 219 | 218 | 519 | 501 | 500 | 478 | 477 | 459 | 261 | 260 | 245 | 184 |
| (E)-2,3-dihydroxypropyl octadec-6-enoate | 357.2996 | 7.98 | 357.2994 | 8.07 | 339 | 283 | 265 | 247 | 177 | 163 | 151 | 149 | 135 | 121 | 339 | 283 | 265 | 247 | 177 | 165 | 163 | 149 | 135 | 121 |
| Cyclo (L-Pro-L-Leu) | 211.1437 | 1.89 | 211.1437 | 1.74 | 211 | 194 | 183 | 155 | 138 | 127 | 86 | 70 | | | 194 | 193 | 183 | 170 | 166 | 155 | 138 | 127 | 86 | 70 |
